# miR-195 and miR-549a Are Essential Biomarkers for Early-Onset Colorectal Cancer

**DOI:** 10.3390/ijms27031379

**Published:** 2026-01-30

**Authors:** Jossimar Coronel-Hernández, Frida Rodríguez-Izquierdo, Berenice Carbajal-López, Eduardo O. Madrigal-Santillán, José Antonio Morales-González, Ayelén Xicohtencatl-Muñoz, Carlos Perez-Plasencia, Claudia M. García-Cuellar, German Calderillo-Ruiz, Yesennia Sánchez-Pérez

**Affiliations:** 1 Subdirección de Investigación Básica, Instituto Nacional de Cancerología, San Fernando No. 22, Tlalpan, Ciudad de México 14080, Mexico; jossithunders@gmail.com (J.C.-H.); ayelenxm@gmail.com (A.X.-M.); 2 Posgrado en Biología Experimental, DCBS, Universidad Autónoma Metropolitana, Iztapalapa, Ciudad de México 09340, Mexico; fridaizquierdo19@gmail.com; 3 Unidad de Biomedicina, Facultad de Estudios Superiores Iztacala, Universidad Nacional Autónoma de México (UNAM), Tlalnepantla 54090, Mexico; carlos.pplas@gmail.com; 4 Unidad Funcional de Gastroenterología, Instituto Nacional de Cancerología, Av. San Fernando 22, Belisario Domínguez Sección 16, Tlalpan, Ciudad de México 14080, Mexico; eb.carbajalopez@gmail.com; 5 Laboratorio de Medicina de la Conservación, Escuela Superior de Medicina, Instituto Politécnico Nacional, Ciudad de México 11340, Mexico; eomsmx@yahoo.com.mx (E.O.M.-S.); jmorales101@yahoo.com.mx (J.A.M.-G.); 6 Dirección de Investigación, Instituto Nacional de Cancerología (INCan), San Fernando No. 22, Tlalpan, Ciudad de México 14080, Mexico; cgarciac@incan.edu.mx

**Keywords:** early-onset colorectal cancer, young, ncRNAs, miRNAs, angiogenesis, migration

## Abstract

Colorectal cancer (CRC) is one of the leading causes of mortality worldwide, with rising cases in individuals under 50 years old, classified as early-onset CRC (EO-CRC). EO-CRC is characterized by having clinical features related to a worse prognosis and outcome. This underscores the critical need for early detection biomarkers. ncRNAs emerge as potential biomarkers for diagnosis, prognosis, and treatment response in other types of cancers. Sequencing data from the NCBI Bioproject PRJNA787417 were analyzed to identify differentially expressed miRNAs in early- and late-onset colorectal cancer (EO-CRC and LO-CRC). Differential expressions were assessed with a log fold change threshold of 1 and an adjusted p-value of 0.05. Predicted mRNA targets were identified via ENCORI and analyzed for pathway enrichment using the SHINYGO algorithm. RNA-seq analysis identified a 25-ncRNA EO-CRC signature, including hsa-miR-195 (downregulated) and hsa-miR-549a (upregulated), with enrichment analyses suggesting associations with MAPK, PI3K, VEGF, and KRAS pathways commonly linked to angiogenesis, migration, and invasion. This preliminary report highlights a 25-gene deregulated signature in EO-CRC, in which hsa-miR-195 and hsa-miR-549a emerge as biomarkers of clinical relevance, regulating key genes involved in angiogenesis, migration, and invasion. Their dysregulation could contribute to the aggressive clinical features and poor outcomes observed in EO-CRC.

## 1. Introduction

Colorectal cancer (CRC) is one of the leading causes of morbidity and mortality worldwide, with a rising incidence in adults under 50 years old, classified as early-onset CRC (EO-CRC). It is projected that the incidence of colorectal cancer (CRC) among young adults in the United States will rise by 1.1% annually for colon tumors and 1.7% for rectal tumors, resulting in 10% of all colon cancers and 22% of all rectal cancers being diagnosed in this demographic by 2030 [1]. EO-CRC more commonly presents distinct clinical features, such as left-sided and rectal distribution, high proportion of mucinous and signet ring cells, poorer cell differentiation, and peritoneal metastasis [2,3]. These aggressive features are linked to a markedly lower survival rate in this population. In 2023, the Gastroenterology Department at Mexico’s National Cancer Institute reported that 30% of deaths caused by colorectal cancer occurred among younger patients [4], highlighting the necessity of identifying molecular biomarkers for early detection in this population.

Several germline and somatic mutations have been identified in CRC patients, with *APC*, *MSH6*, *KRAS*, *TP53*, *BRAF*, and *SMAD4* being among the most prevalent genetic alterations. However, although EO-CRC displays more aggressive clinicopathological features than cases diagnosed at older ages, current evidence indicates that their somatic and germline mutational profiles are largely similar between them and therefore do not adequately explain the differences in tumor behavior [5]. This provides an opportunity to investigate additional molecular mechanisms and to identify novel non-genetic biomarkers that may better explain EO-CRC biology.

Previous studies have demonstrated that non-coding RNAs (ncRNAs), specifically microRNAs (miRNAs) and long non-coding RNAs (lncRNAs), regulate key molecular processes and exhibit differential expressions in cancer patients. Consequently, ncRNAs have been proposed as potential biomarkers for diagnosis, prognosis, and treatment response in several types of cancer. For prostate cancer, PCA3 is the first lncRNA approved by the FDA (Food and Drug Administration) as a diagnostic biomarker more specific than the prostatic antigen [6]. In the EO-CRC context, several miRNA signatures have been proposed. For instance, hsa-miR-193a-5p, hsa-miR-210, hsa-miR-513a-5p, and hsa-miR-628-3p have been shown to diagnose colorectal cancer in young adults with high precision through liquid biopsy, with their expression decreasing after surgery—underscoring their diagnostic and prognostic potential [7]. These findings emphasize the need to develop additional biomarkers. Therefore, the aim of this preliminary report is to present early bioinformatic evidence supporting a 25-gene deregulated signature in EO-CRC, from which hsa-miR-195 and hsa-miR-549a showed particularly relevant clinical associations, as recognized at the ESMO Gastrointestinal Congress 2025 [8]. By sharing these initial results, we seek to emphasize the potential impact of these molecules and encourage their further investigation.

## 2. Results

**Figure 1:**
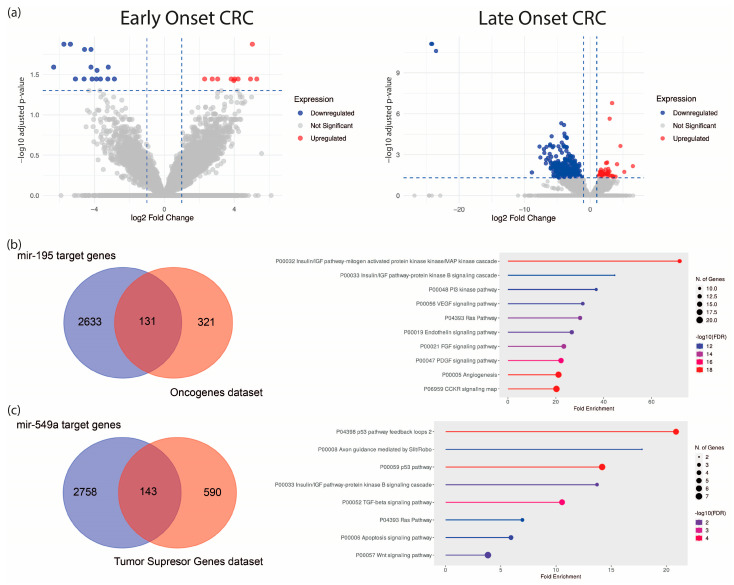
Differential expression analysis of ncRNAs in EO-CRC and LO-CRC. (**a**) Volcano plot of dysregulated ncRNAs in both groups. (**b**) Predicted hsa-miR-195 targets intersecting with tumor suppressor genes and their enriched pathways. (**c**) Predicted hsa-miR-549a targets intersecting with oncogenes and associated oncogenic pathways.

To establish a specific molecular signature of EO-CRC, we analyzed an RNA-seq dataset from eight EO-CRC and five LO-CRC patients (Bioproject PRJNA787417). Differential expression analysis revealed 25 dysregulated ncRNA genes in EO-CRC and 451 in LO-CRC (normalized to their respective age-matched healthy control groups), suggesting distinct ncRNA expression profiles between both groups, with greater dysregulation in older patients (Figure 1a, Table 1 and Table 2, and Supplementary Table S1). To refine the EO-CRC-specific signature, we used a Venn diagram to distinguish ncRNAs unique to EO-CRC from those shared with LO-CRC. This analysis identified 21 ncRNAs exclusive to EO-CRC and four shared between both groups, which are hsa-miR-195, hsa-miR-549a, GAS5, and a novel transcript sense-intronic to GK. Notably, hsa-miR-195 was downregulated, while hsa-miR-549a was upregulated. Among the identified signatures, hsa-miR-195 and hsa-miR-549a emerged as the only miRNAs, and their shared dysregulation across EO-CRC and LO-CRC suggests that they could represent conserved regulatory molecules potentially involved in early colorectal tumorigenesis across age groups.

To explore their functional relevance, we predicted hsa-miR-195 and hsa-miR-549a target genes using the ENCORI algorithm, identifying 2764 and 2901 predicted targets, respectively. These were cross-referenced via a Venn diagram with oncogene and tumor suppressor gene datasets (OncoKB™ Cancer Gene List), respectively. The resulting genes were subjected to gene set enrichment analysis, which implied an association with major oncogenic pathways, including MAPK, PI3K, VEGF, and KRAS (Figure 1b,c).

**Figure 2:**
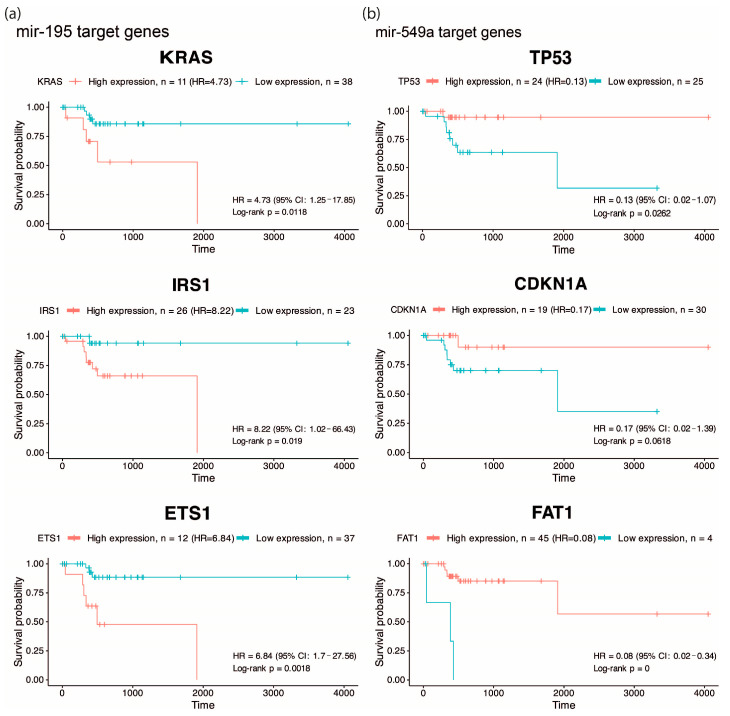
Overall survival analysis of predicted targets. Kaplan–Meier curves showing overall survival according to the expression of KRAS, IRS1, and ETS1 (predicted targets of hsa-miR-195) (**a**), and TP53, CDKN1A, and FAT1 (predicted targets of hsa-miR-549a) (**b**) in EO-CRC patients, highlighting their association with poor prognosis..

One of the most important features of EO-CRC is its aggressive clinical presentation at diagnosis, frequently associated with peritoneal metastasis. Accordingly, we focused on pathways related to angiogenesis, migration and cellular invasion, and by overall survival analysis, we found that high expression of KRAS, IRS1, ETS1 (targets of hsa-miR-195, Figure 2a) and low expression of TP53, CDKN1A and FAT1 (targets of hsa-miR-549a, Figure 2b) were associated with low survival (HR: 4.73, 8.22, 6.84, 0.13, 0.17 and 0.08), suggesting that hsa-miR-195 and hsa-miR-549a target genes may serve as potential prognostic biomarkers in CRC. Altogether, these findings highlight hsa-miR-195 and hsa-miR-549a as promising regulators of key oncogenic pathways in CRC.

## 3. Discussion

The present study provides an initial characterization of ncRNA dysregulation in EO-CRC based on a focused, well-defined cohort, identifying molecules with potential clinical relevance that warrant further evaluation in larger and independent populations. In this context, our results highlight miR-195 and miR-549a as clinically relevant biomarkers in EO-CRC. Consistent with previous reports, miR-195 downregulation has been associated with CRC progression, in part through the dysregulation of oncogenic pathways such as Wnt/β-catenin signaling, a molecular axis frequently altered in early-onset disease [9,10,11]. Other studies have indicated that Kras and PIK3 signaling pathways are essential to EO-CRC establishment [5,12], which are predicted to be targeted genes of miR-195 (Figure 1b,c). In the KrasG12D-Cre mouse model, miR-195 levels were reduced at 10 weeks of age; however, at 30 weeks of age, miR-195 restored basal levels, suggesting that downregulation of this miRNA and Kras mutation could be necessary to allow early tumor initiation [13]. In addition, in endothelial cells, breast cancer, and metabolic disease, the miR-195/IRS1 axis is described as a regulator of PI3K/AKT pathways, promoting tumor growth and tissue remodeling when miR-195 is downregulated [14,15,16]. For instance, in gastric cancer, miR-195-5p was shown to suppress ETS1, a known oncogenic factor whose elevated expression promotes proliferation and invasion [17]. Furthermore, there is evidence about miR-195’s relevance in clinical studies. Its low expression is associated with poor outcomes in 140 CRC formalin-fixed paraffin-embedded samples [18]; further, in CRC plasma, the miR-195 levels were significantly lower than in healthy control plasma, emerging as a potential CRC biomarker [19,20].

On the other hand, miR-549a is poorly studied in cancer and other diseases; it has been investigated for its function as a circulating diagnostic biomarker for multiple sclerosis, but without significant results [21]. Additionally, it has been studied as a prognostic/predictive biomarker in locally advanced adenocarcinomas of the gastroesophageal junction, where its overexpression was correlated with a positive therapy response [22]. Meanwhile, an advanced breast cancer clinical study indicated that miR-549a overexpression was negatively associated with clinical outcome in 59 fulvestrant-treated patients [23]. Specifically, in CRC, there is only one study that reveals that elevated levels of miR-549a in CRC tissue and plasma were associated with the invasion of tumor depth (*p* = 0.044), and according to the results of ROC analysis, miR-549a may serve to diagnose CRC [24]. Although miR-549a has been less explored in colorectal cancer, one of its predicted targets, FAT1, emerges as a molecule of relevance. In our analysis, low FAT1 expression was strongly associated with reduced overall survival, suggesting a potential tumor-suppressive role in EO-CRC (Figure 2b). This observation aligns with genomic studies reporting that FAT1 frequently harbors mutations in EO-CRC, including cohorts of African, Asian, and Western patients [25,26,27,28]. Given that loss-of-function mutations in FAT1 can impair its regulatory activity, the recurrent alterations described in these studies raise the possibility that genomic disruption of FAT1 may contribute to its reduced expression, thereby amplifying the deleterious effects predicted from miR-549a-mediated regulation.

Sequencing genomic studies indicate that the mutational landscape of colorectal cancer (encompassing genes such as *APC*, *MSH6*, *KRAS*, *TP53*, *BRAF*, and *SMAD4*) does not substantially differ between EO-CRC and LO-CRC [5]. This has raised growing interest in non-genetic regulation, including epigenetic and post-transcriptional mechanisms, as potential contributors to the aggressive clinical phenotype of EO-CRC. In this context, reduced miR-195 expression in CRC has been reported to be regulated through competing endogenous RNA mechanisms involving several lncRNAs (SNHG1, AFAP1-AS1, and PEG13) as well as circRNAs (circSEMA5A, circ_0060927, and circRASSF2) [11,29,30,31]. Moreover, growing evidence suggests that environmental exposures may contribute to EO-CRC through microbiome-associated and epigenetic mechanisms capable of modulating ncRNA regulatory networks, highlighting the importance of future investigations aimed at elucidating how microbiome alterations influence EO-CRC development in young populations [32].

## 4. Materials and Methods

### 4.1. ncRNA Identification

To identify differentially expressed ncRNAs between patients with EO-CRC and late-onset colorectal cancer LO-CRC, sequencing data were accessed from the NCBI bioproject PRJNA787417 [33]. The data set included samples from seven young healthy patients (ages 32–47), eight young patients with colorectal cancer (ages 33–47), five late healthy patients (ages 60–72), and five patients with late colorectal cancer (ages 60–64). Raw sequencing data were downloaded in FASTQ format using the Sequence Read Archive (SRA) with applied trimming and filtering. Contaminating 3′ adapter sequences (TGGAATTCTCGGGTGCCAAGG) were removed using Cutadapt (v.5.0) within the SystemPipe framework parameters, m, q20, and trim-n, ensuring high-confidence reads that are suitable for the downstream analysis. Sequence alignment was performed with Bowtie2 (Jhons Hopkins University, Baltimore, MD, USA, v.2.5.4) against the ENSEMBL Index of non-coding RNAs, corresponding to Homo_sapiens.GRCh38.113.chr.gtf, with the parameters-verysensitive and -N1 (allows maximum 1 mistmatch). The resulting SAM files were converted to BAM using Rsamtools (v.2.20.0), and alignment counts (excluding genomic mapping) were generated with this same package. Finally, differential expression analysis was performed using DESeq2 (v.1.44.0), comparing CRC patients with age-matched healthy controls (EO-CRC vs. young healthy controls, and LO-CRC vs. late healthy individuals), the threshold considered for differential expression was log2 fold change > 1 or <−1, and an adjusted *p*-value of <0.05. Based on these results, we selected only miR-195 and miR-549a for subsequent analysis.

### 4.2. mRNA Target Prediction and Pathway Enrichment Analysis

To predict the mRNA targets of miR-195 and miR-549a, we employed the ENCORI (Yang Lab, Xiamen, China, starBase v2.0) algorithm, and as selection criteria, the interactions of miRNA-target were performed by multiple target-predicting programs (PITA, miRanda, PicTar, and TargetScan). Due to their level of expression, we considered miR-195 as a tumor suppressor miR and miR-549a as an oncomiR. Thus, by a Venn diagram, we compared the predicted target genes for each miRNA with an oncogene and tumor suppressor gene dataset to obtain the curated target genes, which were loaded into Shiny GO (South Dakota State University, Brookings, SD, USA, v.0.85.1) gene-set enrichment tool using a 0.05 *p*-value cutoff of FDR.

### 4.3. Survival Analysis

Clinical and RNAseq data corresponding to the TCGA colon and rectal adenocarcinoma cohorts were downloaded using the Bioconductor TCGAbiolinks package (v.2.32.0). A total of 49 colorectal cancer patients aged ≤ 50 years of age, with complete clinical and expression data, were included in the survival analysis. The median follow-up duration was 457 days (range: 0–4051 days), during which nine death events were recorded. The expression of each gene was evaluated as a continuous variable and divided into high and low expression groups using the survminer package (v.0.5.1). Kaplan–Meier curves were compared between groups using a log-rank test using the R (RStudio Inc., Boston, MA, US, v.4.0.1+748) survival package (v.3.8.3).

## 5. Conclusions

This preliminary analysis reveals a 25-ncRNA signature in EO-CRC and identifies miR-195 and miR-549a as pivotal molecules with potential clinical relevance. The biological evidence supporting miR-195 loss and miR-549a overexpression provides a plausible molecular framework for the aggressive behavior observed in young patients. These findings, presented at the ESMO Gastrointestinal Congress 2025, highlight the promise of these miRNAs as candidate non-invasive biomarkers, whose clinical utility will require further validation in independent cohorts and liquid biopsy-based studies.

## Figures and Tables

**Table 1:** Up- and downregulated ncRNAs in EO-CRC. Values are expressed in log2 FoldChange. In red, miRNAs shared between EO-CRC and LO-CRC are highlighted.

Early Onset Colorectal Cancer							
Down (logFC < −1, *p* adj < 0.05)				Up (logFC > 1, *p* adj < 0.05)			
Name	ENSEMBL ID	Type	log2 FC	Name	ENSEMBL ID	Type	log2 FC
MIR195	ENST00000385194	miRNA	− 3.24	RN7SK	ENST00000365097	Misc RNA	4.93
sense intronic to GK-	ENST00000497961	lncRNA	−5.75	MIR549A	ENST00000385268	miRNA	5.03
FXYD6-AS1	ENST00000534150	lncRNA	−3.65	novel transcript	ENST00000588088	lncRNA	2.30
LINC02169	ENST00000649252	lncRNA	−4.14	CSNK2A2-AS	ENST00000660742	lncRNA	3.97
novel transcript	ENST00000651076	lncRNA	−4.57	LINC02003	ENST00000664653	lncRNA	4.22
novel transcript	ENST00000663875	lncRNA	−4.20	SULT2B1-AS	ENST00000666424	lncRNA	3.05
novel transcript	ENST00000666596	lncRNA	−2.86	CHROMR	ENST00000686412	lncRNA	2.74
ERBIN-AS	ENST00000741190	lncRNA	−5.38	novel transcript	ENST00000771184	lncRNA	5.28
SNHG1	ENST00000742646	lncRNA	−4.59	LINC01210	ENST00000774633	lncRNA	4.02
LIPE-AS1	ENST00000750104	lncRNA	−6.33	MTHFD2L-AS	ENST00000806141	lncRNA	3.82
novel transcript	ENST00000785120	lncRNA	−3.85				
THRB-AS1	ENST00000807235	lncRNA	−5.09				
GJD2-DT	ENST00000808234	lncRNA	−4.20				
GAS5	ENST00000828023	lncRNA	−3.89				
GAS5-372	ENST00000828028	lncRNA	−3.21				

**Table 2:** Up- and downregulated ncRNAs in LO-CRC. Summary of the 10 most overexpressed and downregulated ncRNAs in LO-CRC patients. The complete list comprising all 451 ncRNAs is provided in the Supplementary Materials.

Late Onset Colorectal Cancer							
Down (logFC < −1, *p* adj < 0.05)				Up (logFC > 1, *p* adj < 0.05)			
Name	ENSEMBL ID	Type	log2 FC	Name	ENSEMBL ID	Type	log2 FC
novel transcript	ENST00000629772	miRNA	−8.89	MIR1269A	ENST00000408636	miRNA	6.52
SOX21-AS1	ENST00000690218	lncRNA	−6.99	SMIM2-AS1	ENST00000762356	lncRNA	5.21
novel transcript	ENST00000809922	lncRNA	−6.76	novel transcript	ENST00000563162	lncRNA	4.61
novel transcript	ENST00000747975	lncRNA	−6.71	MIR549A	ENST00000385268	miRNA	4.10
MIR103B	ENST00000636572	miRNA	−6.11	novel transcript	ENST00000418585	lncRNA	3.88
PPP1R13B-DT	ENST00000654695	lncRNA	−6.11	novel transcript	ENST00000822100	lncRNA	3.48
FAM83C-AS1	ENST00000765530	lncRNA	−6.10	MIR135B	ENST00000362189	miRNA	3.35
novel transcript	ENST00000720368	lncRNA	−6.03	novel transcript	ENST00000790582	lncRNA	3.34
novel transcript	ENST00000831016	lncRNA	−6.00	GUSBP11-465	ENST00000735274	lncRNA	3.06
MIR378A	ENST00000362177	miRNA	−5.88	MIR224	ENST00000384889	miRNA	2.97
MIR195	ENST00000385194	miRNA	− 2.78				

## Data Availability

The original contributions presented in this study are included in the article/Supplementary Material. Further inquiries can be directed to the corresponding authors.

## Supplementary Materials

The following supporting information can be downloaded at: https://www.mdpi.com/article/10.3390/ijms27031379/s1.
